# 

**Published:** 1881-07-20

**Authors:** 


*Lactopeptine* is a very valuable remedy in cholera infantum and the diarrhœas of children, especially in any case dependent upon or connected with indigestion.

---

*Lambert & Co.* have kindly sent us samples of their preparation Listerine. The formula gives promise of excellent results from this article in the dressing of wounds, etc. It is likely to prove a great convenience to both surgeon and physician as a purifying and antiseptic article presented in a very convenient form.

---

DR. W. D. BIZZELL, formerly of the Mobile Medical College, has been elected to the chair of Chemistry in the Southrrn Medical College, in place of E. J. Hallock, resigned.

The officers elect of the Faculty for the year 1881 and '82 are as follows:

Prof. T. S. POWELL, President.
Prof. R. C. WORD, Vice-President.
Prof. W. P. NICOLSON, Dean.
MR. J. J. TOON, Treasurer.

The Annual Announcement for 1881 and 1882 is now ready.

